# Myeloid Derived Suppressor Cells Expansion Persists After Early ART and May Affect CD4 T Cell Recovery

**DOI:** 10.3389/fimmu.2019.01886

**Published:** 2019-08-08

**Authors:** Chiara Agrati, Nicola Tumino, Veronica Bordoni, Carmela Pinnetti, Andrea Sabatini, Alessandra Amendola, Isabella Abbate, Patrizia Lorenzini, Annalisa Mondi, Rita Casetti, Eleonora Cimini, Germana Grassi, Andrea Antinori, Alessandra Sacchi

**Affiliations:** ^1^Cellular Immunology Laboratory, National Institute for Infectious Diseases Lazzaro Spallanzani-IRCCS, Rome, Italy; ^2^Virology Laboratory, National Institute for Infectious Diseases Lazzaro Spallanzani-IRCCS, Rome, Italy; ^3^Clinical Division, National Institute for Infectious Diseases Lazzaro Spallanzani-IRCCS, Rome, Italy

**Keywords:** MDSC, HIV, early ART, early T cell progenitors, TRAIL, GM-CSF

## Abstract

Myeloid-derived suppressor cells (MDSC) are expanded during HIV-1 infection and correlated with disease progression. MDSC expand in the early phase of primary infection depending on TRAIL level. In this study we evaluated the effect of ART on the frequency of MDSC in patients with primary HIV infection (PHI), and their impact on CD4 T cell reconstitution. MDSC frequency was evaluated by flow-cytometry in 60 PHI patients at 12, 24 and 48 weeks after ART initiation. Cytokine plasma levels were evaluated by Luminex technology at the same time points. The capacity of MDSC to modulate hematopoietic early progenitor cells' expansion was evaluated using the OP9/Dl1 *in vitro* system. As previously described, polymorphonuclear-MDSC (PMN-MDSC) frequency was higher in PHI compared to healthy donors. Interestingly, 48 weeks of successful ART failed to normalize the PMN-MDSC frequency. Moreover, PMN-MDSC frequency was not correlated with residual viral load, suggesting that the persistence of PMN-MDSC was not due to residual viral replication. Interestingly, patients with low PMN-MDSC frequency (<6%) at T0 had a higher HIV DNA at the same time point than individuals with high PMN-MDSC frequency (>6%). We also found an inverse correlation between PMN-MDSC frequency and CD4-T cell count at 48 weeks post-ART, which was confirmed by multivariate analysis adjusting for age and CD4 T cell number at baseline. These data suggest that the persistence of PMN-MDSC may impact CD4 T cell recovery. Indeed, *in vitro* PMN-MDSC impaired the expansion of CD34+CD38- hematopoietic early progenitors. Further, a balance between TRAIL and GM-CSF may be necessary to maintain a low MDSC level. In conclusion, early ART initiation was not able to normalize PMN-MDSC frequency that might impact the CD4 T cell recovery. These data open new questions regarding the clinical impact of MDSC persistence in HIV+ patients, in particular on non-AIDS related diseases.

## Introduction

Several conditions associated with various types of chronic inflammation result in aberrant and sustained myelopoiesis, characterized by the accumulation of myeloid cells with regulatory functions and ultimately defined as myeloid-derived suppressor cells (MDSC) (1–3). The first observation of myeloid cells with suppressive functions was reported 20 years ago in cancer-bearing mice (4). However, the importance of this cell population has only recently been pointed out, due to accumulating evidence on its contribution to the negative regulation of immune responses during cancer and other diseases. It has been shown that MDSC are a heterogeneous cell population at different stages of differentiation, comprising myeloid cell progenitors and mature cells (5). In humans, two major MDSC subsets have been identified, the monocytic-MDSC (Mo-MDSC) and polymorphonuclear-MDSC (PMN-MDSC) subsets, expressing low level of HLA-DR, myeloid markers as CD11b and CD33, and CD14 or CD15 respectively (6). A third immature subset of MDSC (immature-MDSC, i-MDSC) comprises cells with immaturity characteristics and is defined essentially as lineage negative cells (7). Suppressive functions are mediated by several major molecular pathways, including inducible nitric oxide (NO) synthetase (iNOS), arginase-1 (Arg-1), NADPH oxidase (NOX2), and TGFβ (6). MDSC are able to inhibit a broad range of immune cell functions such as T cell proliferation and activation (8, 9), cytokine production by macrophages (10), and NK cell function (11). They are also involved in the induction of regulatory T cells (12), thus playing a pivotal role in regulating immune response. It has been reported that MDSC increase in several acute and chronic infections [reviewed in (13)]. We and others showed the expansion of Mo-MDSC and PMN-MDSC during chronic HIV infection (14–16), which correlated with CD4 T cell number and viral load. In HIV-infected patients, MDSC have a potent inhibitory capacity toward HIV-specific CD8 T cell response, thus participating in the maintenance of HIV persistence. More recently, we observed the spread of suppressive PMN-MDSC in the acute phase of HIV infection (17), in particular in the first Fiebig stages. Furthermore, PMN-MDSC expansion during primary HIV infection may be, at least in part, regulated by tumor necrosis factor-related apoptosis-inducing ligand (TRAIL), which is able to limit PMN-MDSC survival in the absence of pro-survival factors as GM-CSF (17, 18). However, different issues on MDSC during HIV infection remain still unclear, in particular whether the inhibition of HIV replication by ART directly induces MDSC constriction. A reduction in MDSC frequency has been observed after 3 months of therapy in a small group of chronically infected patients (14), but no data are available for longer treatment. The aim of the present work was to better understand the dynamic of MDSC during ART started in the early phase of HIV infection and the possible impact of their persistence on immune reconstitution.

## Methods

### Study Population

Patients with primary HIV infection (PHI, *n* = 60) were enrolled at the National Institute for Infectious Diseases (INMI) “Lazzaro Spallanzani” (Rome, Italy). At baseline the median CD4 T cell number was 570.5 (IQR 427.8–731.5), and median viral load (VL) was 5.3 Log copies/ml (IQR 4.31–6.26). Healthy individuals (HD, *n* = 23) were included as controls. Baseline patients' characteristics are summarized in Table 1. PHIs were recruited before therapy initiation (T0), 12, 24, and 48 weeks (T12, T24, T48) after ART onset. After 48 weeks of therapy, median CD4 T cell number was 806 (IQR 623-997), and median VL <40 copies/ml (range: not detected- <40 copies/ml). The study was approved by the Institutional Review Board of the INMI “Lazzaro Spallanzani” (ALPHA and SIREA studies) and signed written informed consent was obtained from all patients in accordance with the Declaration of Helsinki.

**Table 1:** Study participants.

**Characteristics**	**PHI**	**HD**
	***n* = 60**	***n* = 23**
**STATUS**		
Naive	60 (100%)	na
Female gender, *n*(%)	5 (8.3%)	12 (52%)
Age, years, median (IQR)	35 (27.8–41)	37 (27-48)
**ETHNICITY**, ***n*****(%)**		
Caucasian	57 (95%)	23 (100%)
African	0 (0%)	0 (0%)
Hispanic	1 (1.7%)	0 (0%)
Asian	2 (3.3%)	0 (0%)
**MODE OF HIV TRANSMISSION**		
heterosexual	12 (20.0%)	na
homosexual	47 (78.3%)	na
Other/unknown	1 (1.7%)	na
**FIEBIG STAGE**		
II/III	12 (20.0%)	na
IV	15 (25.0%)	na
V/VI	31 (51.7%)	na
Unknown	2 (3.3%)	na
CD4 at baseline, median (IQR)	570.5 (427.8–731.5)	na
**HIV-RNA AT BASELINE, COPIES/mL**		
not detected	0 (0%)	na
<40 detected	1 (1.7%)	na
≥40 and <1,000	1 (1.7)	na
≥1,000	53 (88.3%)	na
>10,000,000	5 (8.3%)	na
**ART REGIMEN**		
TDF+FTC+DRV/RTV+RGV	39 (65.0%)	na
TDF+FTC+DTG	18 (30.0%)	na
TDF+FTC+RPV	1 (1.7%)	na
DRV/RTV+RGV	1 (1.7%)	na
TDF+FTC+DRV/RPV	1 (1.7%)	na

*na, not applicable; TDF, Tenofovir; FTC, emtricitabine; DRV, Darunavir; RTV, Ritonavir; RGV, raltegravir; DTG, dolutegravir; RPV, Rilpivirine*.

### PBMC Separation and Stimulation

Peripheral blood mononuclear cells (PBMCs) were isolated from peripheral blood by density gradient centrifugation (Lympholyte-H; Cederlane). After separation, PBMCs were resuspended in RPMI 1640 (EuroClone) supplemented with 10% heat-inactivated fetal bovine serum (FBS) (EuroClone), 2 mmol/L L-glutamine, 10 mmol/L HEPES buffer (N-2-hydroxyethylpiperazine-N-2-ethane sulfonic acid), 2 mmol/L penicillin, and 50 μg/mL streptomycin (EuroClone), hereafter termed R10. HIV-specific CD8 T cell response after 48 weeks was evaluated by stimulating PBMCs with a pool of Gag-, Nef-, and Tat-derived peptides (1 ug/mL each peptide; HIV/AIDS Reagent Program, Germantown, MD). Brefeldin A was added after 1 h, and IFN-γ production was tested by flow cytometry after 18 h.

### Flow Cytometry

Evaluation of MDSC percentage was accomplished with 0.5 × 10^6^ PBMCs stained with anti-CD15 FITC, anti-CD33 PE, anti-HLA-DR PERCP, cocktail of antibodies anti-CD3, -CD56, -CD19 (Lin) APC, anti-CD14 APC-H7, anti-CD11b PE-C7, anti-CD16 Pacific Blue (BD Biosciences). The frequency of hematopoietic progenitors was evaluated on 1 × 10^∧^^5^ viable cells by using Lineage cocktail 1 FITC, anti-CD34 APC, anti-CD45 V500, 7AAD (BD Biosciences), and anti-CD38 PE-Vio770 (Miltenyi Biotec). Intracellular flow cytometry was performed by using anti-CD3, anti-CD8, and anti-IFN-γ (BD Biosciences, USA). Acquisition of 100,000 events was performed in the leukocyte-gated population on FACS CANTO II and analyzed with FACS DIVA software (BD Biosciences).

### PMN-MDSC Purification

PMN-MDSC were purified from PBMC by immunomagnetic sorting by using CD15 microbeads (Miltenyi Biotec) following the manufacturer's procedure. The purity of PMN-MDSC was >90% as verified by flow cytometry (data not shown). Purified cells were rested for 18 h in R10.

### Residual Viral Load and HIV DNA Quantification

Residual viral load (RVL) was quantified with the ultrasensitive protocol of the Abbott RealTime HIV-1 assay (LLOD: 5 copies/ml), obtained applying the following modifications to the standard procedure (19): (1) higher sample volume (3.2 ml) concentrated by ultracentrifugation; (2) calibration curve extended toward HIV-RNA lower levels; (3) reduced volume of internal control; (4) “open” software. Each patient underwent multiple quantifications of RVL.

Total HIV-DNA was quantified in PBMCs collected by density gradient centrifugation as previously reported. Automated DNA extraction from PBMC was performed on the QIAsymphony platform using an inhouse modification of the DSP Virus/Pathogen Midi Kit protocol (Qiagen, Milan Italy). A Real-Time PCR targeting HIV-LTR was used to quantify total HIV-1 DNA as previously described (20), whereas another Real-Time PCR targeting the housekeeping gene hTERT was performed to refer HIV-DNA copies to a million PBMCs (21) on LightCycler 2.0 platform (Roche, Monza Italia).

### Hematopoietic Progenitor Cells Co-culture

The impact of MDSC on bone marrow-CD34+ hematopoietic progenitor cells differentiation toward T cells was evaluated by culturing bone marrow-CD34+ hematopoietic progenitor cells with OP9-DL1 stromal cells for 9 days as previously described (22). Briefly, 50 × 10^∧^^4^ purified bone marrow-CD34+ cells (from Stem Cell Technologies) were co-cultured on OP9-DL1 cells seeded in a 24-well plate in alpha-minimal essential medium (Gibco, Grand Island, NY) supplemented with 20% fetal bovine serum in the presence of interleukin IL-7 (1 ng/ml; Immunotools, Friesoyte, Germany), Stem Cell Factor (SCF, 30 ng/ml; ENZO Life Science Farmingdale, NY), and FMS-like tyrosine kinase 3 (FLT3) ligand (5 ng/ml; PeproTech, Inc., Rocky Hill, NJ). Purified PMN-MDSC from PHI were added at different ratios (BM-CD34: PMN-MDSC 1:0, 1:1, 1:3). After 9 days of culture, the cells were filtered 40 μm (BD, Falcon) and CD34+CD38- progenitors were evaluated by flow-cytometry.

### Plasma Cytokine Levels

Plasma samples were obtained after speed centrifugation for 10 min at 2,000 rpm and immediately stored at −80°C. Plasma samples were assayed by using the multiplex bead-based assays Bio-Plex Pro Human group I 19 plex (IL-1β, IL-2, IL-4, IL-5, IL-6, IL-7, IL-8, IL-9, IL-10, IL-12, IL-13, IL-17, G-CSF, GM-CSF, IFN-γ, MCP-1, MIP1-β, RANTES, TNF-α) and Bio-Plex Pro Human group II cytokines 21 plex (IL-1β,IL-2Rα, IL-3, IL-12, IL-16, IL-18, CTACK, GROα, HGF, IFN-α2, LIF, MCP-3, M-CSF, MIF, MIG, NGF, SCF, SCGF, SDF-1, TNF-β, TRAIL) from BioRad Laboratories. Plates were measured using the Bio-Plex MagPix System and analyzed with the Bio-Plex Manager Version 6.0 (BioRad Laboratories).

### Statistical Analysis

GraphPad Prism version 4.00 for Windows (GraphPad Software) and STATA 15.1 were used to perform statistical analyses. The non-parametric Kruskal-Wallis/Friedman with Dunn's post test were used to compare continuous variables. Correlations were evaluated with the non-parametric Spearman test. Logistic regression analysis was used to estimate OR and 95% CI of factors associated with high/low CD4 cell count at baseline and at 48 w. A *p*-value < 0.05 was considered statistically significant.

## Results

### ART During PHI Failed to Decrease PMN-MDSC

**Figure 1:**
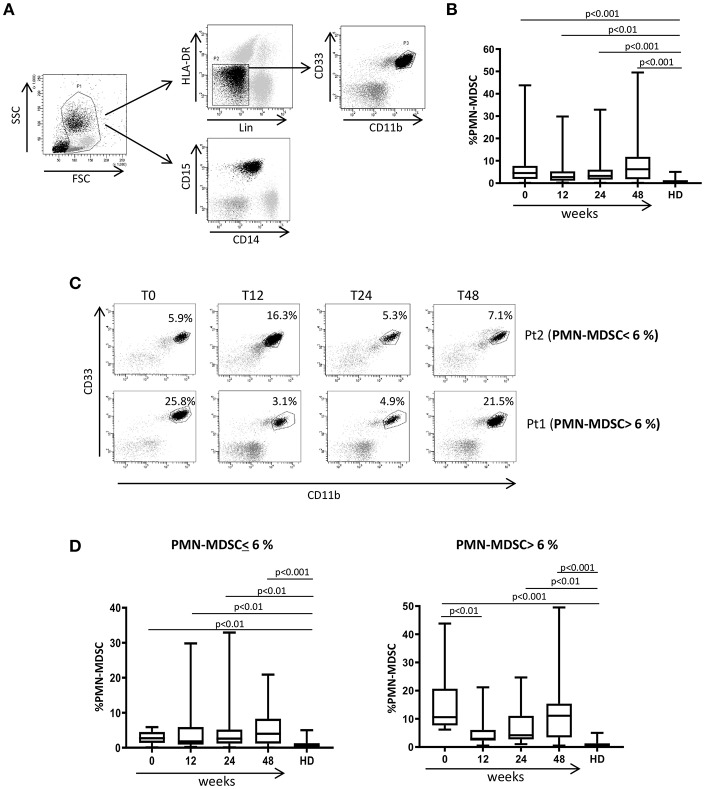
MDSC frequency during ART of primary HIV infection. **(A)** Gating strategy used to identify MDSC: in the morphological gate (FSC/SSC) we excluded debris, then we gated Lin-/HLA DRlow/- cells. In this gate we selected CD11b+CD33+ cells (MDSC). The expression of CD14 and CD15 is shown on cells selected from the morphological gate (P1). The frequency of PMN-MDSC was calculated in P1. **(B)** PMN-MDSC frequency in HD (23), PHI (60) at baseline (0) and after 12, 24, and 48 weeks of ART. The percentage of Lin-/HLA-DR-/CD11b+/CD33+ has been calculated in the P1 gate. **(C)** Representative dot plots from two patients with different initial PMN-MDSC frequencies (<6% and >6%), showing PMN-MDSC frequency (Lin-/HLA DRlow/- /CD11b+/CD33+ cells) at baseline and after 12, 24, and 48 weeks of ART. **(D)** PMN-MDSC frequency at the indicated time points after ART in patients with an initial PMN-MDSC frequency higher (*n* = 20, right panel) and lower (*n* = 40, left panel) than 6%. Results are shown as box and whiskers. The Friedman or Kruskall-Wallis test (with Dunn's multiple comparison post test) were applied.

We previously demonstrated that PMN-MDSC are expanded during PHI (17). We wondered whether successful ART was able to decrease PMN-MDSC frequency. In agreement with previous research, before ART the frequency of PMN-MDSC was higher in PHI than HD. In contrast, Mo-MDSC were not detected in both PHI and HD. After ART initiation, the frequency of PMN-MDSC persisted higher than HD during all time points (Figures 1A,B). Because of the high variability of PMN-MDSC frequency in PHI, we grouped patients based on PMN-MDSC frequency at T0: low PMN-MDSC group (<6%) and high PMN-MDSC group (>6%); we decided to use the 6% cut off since it represented the highest PMN-MDSC frequency found in the HD group. We observed that in the high PMN-MDSC group the frequency of MDSC decreased at T12 compared to T0, but at T24 it went back to a level similar as T0 (Figure 1C Pt1, and Figure 1D left panel). Moreover, at all time points except T12, PMN-MDSC were higher than HD (Figure 1C). Differently, in the low PMN-MDSC group, no modulation of PMN-MDSC frequency was observed, remaining stably higher compared to HD at all time points (Figure 1C Pt2, and Figure 1D right panel).

**Figure 2:**
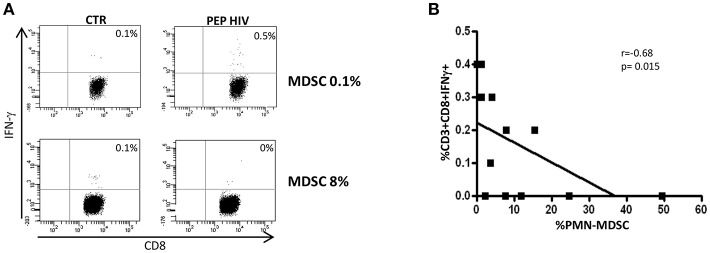
PMN-MDSC maintain inhibitory capacity. **(A)** IFN-γ production by CD8 T cells stimulated with HIV-specific peptides (HIV PEP) or not stimulated (CTR) from two representative PHIs after 48 weeks of ART. The percentage of HIV-specific CD8 T cell response was evaluated in the gate of CD3+CD8+ T cells. **(B)** Correlation between PMN-MDSC frequency and the percentage of CD8 T cells producing IFN-γ (evaluated by flow cytometry) from 12 PHIs was evaluated by the Spearman test. The *p* < 0.05 was considered statistically significant.

In order to understand whether PMN-MDSC maintain a suppressive capability after 48 weeks from ART initiation, we tested the percentage of HIV-specific CD8 T cells producing IFN-γ in a patient subgroup. We found a lower percentage of cells from patients with high PMN-MDSC frequency than those with low PMN-MDSC (Figure 2A). Accordingly, an inverse correlation was observed between PMN-MDSC frequency and the percentage of CD8 T cells producing IFN-γ after HIV-derived peptide stimulation (Figure 2B). These data suggest that PMN-MDSC retained their suppressive capacity after 48 weeks of therapy.

### Evaluation of the Association Between RVL or HIV Reservoir and PMN-MDSC Frequency

**Figure 3:**
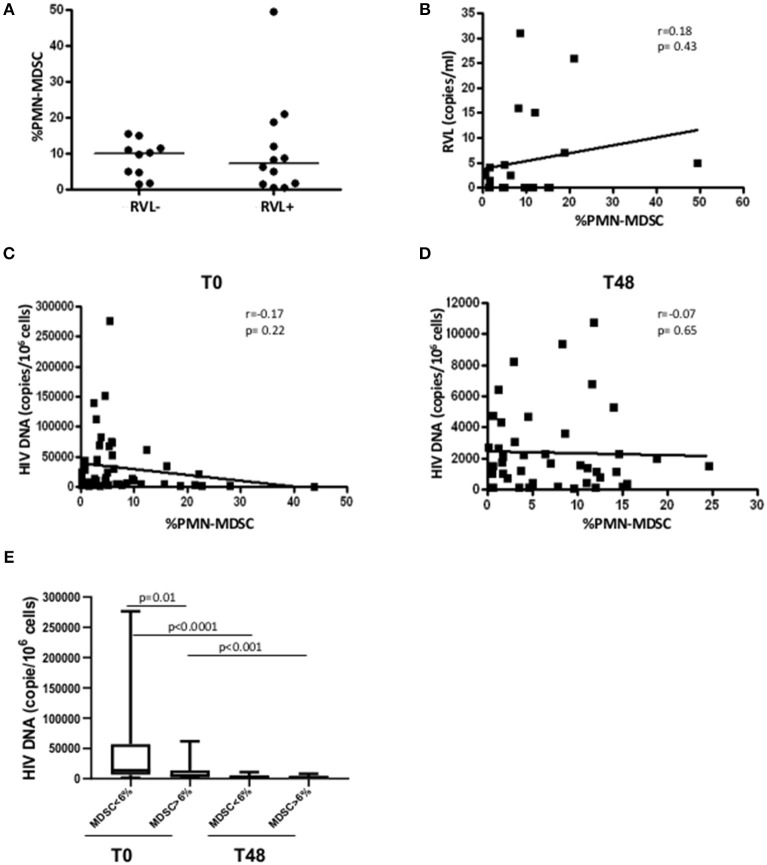
PMN-MDSC frequency is not associated with residual viral load nor with HIV DNA. **(A)** PMN-MDSC frequency in PHI without detectable (RVL-, *n* = 10) and detectable (RVL+, *n* = 12) residual viral load. **(B)** Correlation between PMN-MDSC frequency and residual viral load of 22 PHIs after 48 weeks of ART. Correlation between PMN-MDSC frequency and HIV DNA at T0 **(C)** and after 48 weeks of therapy **(D)**. The Spearman test was applied to evaluate correlations. **(E)** HIV DNA at T0 and T48 in the groups with PMN-MDSC lower and higher than 6% at T0. The Mann Whitney test was used to compare different patient groups and the Wilcoxon test compared different time points. *p* < 0.05 was considered statistically significant.

After 48 weeks of treatments, all enrolled patients had a viral load under the limit of detection (Table 1), indicating that persistent viral replication was not involved in the maintenance of PMN-MDSC. To confirm that viral load was not the direct driving force of PMN-MDSC expansion, we evaluated the RVL after 48 weeks from therapy initiation. We did not find difference in PMN-MDSC frequency between patients with and without detectable RVL (Figure 3A); moreover, no correlation was observed between RVL and PMN-MDSC frequency (Figure 3B). We also evaluated RVL in the low and high PMN-MDSC frequency groups, and no difference was observed.

Since PMN-MDSC increased very early after infection and can inhibit different immune cell functions, we wondered whether they could affect the HIV reservoir establishment. To this aim, we evaluated the correlation between MDSC frequency and HIV DNA before and after 48 weeks of ART. We did not find any correlation between the two variables (Figures 3C,D). However, grouping patients in low and high PMN-MDSC, we found a higher HIV DNA level in the low PMN-MDSC group compared to the high group (Figure 3E), suggesting that PMN-MDSC might interfere with HIV reservoir establishment. After 48 weeks from therapy initiation a decrease of HIV DNA was observed in both groups, and no difference was observed between the two groups at T48 (Figure 3E).

### Effect of PMN-MDSC on Early Progenitor's Expansion During *in vitro* T Cell Differentiation

**Figure 4:**
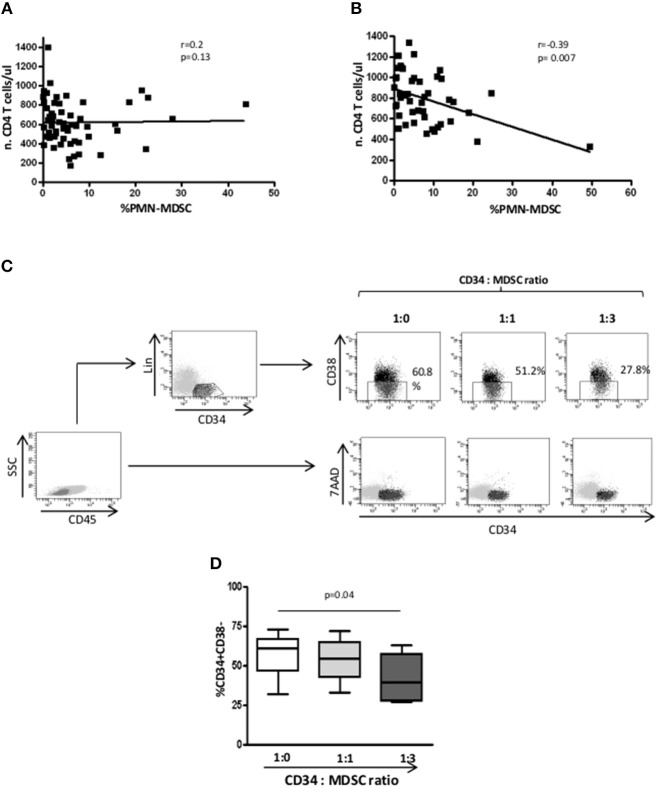
After 48 weeks, PMN-MDSC frequencies correlate with CD4 T cell count. **(A)** Correlation between PMN-MDSC frequency and CD4 T cell count before therapy initiation (*n* = 60). **(B)** Correlation between PMN-MDSC frequency and CD4 T cell count after 48 weeks of therapy (*n* = 50). The Spearman test was applied to evaluate correlations. *p* < 0.05 was considered statistically significant. **(C)** Gating strategy to evaluate the percentage of CD34+CD38- early progenitors. In the gate of CD45+ cells, the hematopoietic CD34+ progenitor cells were selected as Lin-CD34+, and the percentage of CD34+CD38- were evaluated among Lin-CD34+ cells in the indicated conditions. 7AAD expression was evaluated in the gate of CD45+ cells. **(D)** Percentage of CD34+/CD38- cells evaluated by flow cytometry, in the presence of PMN-MDSC (1:0, 1:1, and 1:3 ratios) from 4 PHI. The Friedman test with Dunn's multiple comparison post test was applied.

As previously demonstrated (17), in PHI patients before starting ART, no correlation between PMN-MDSC frequency and CD4 T cell number was observed (Figure 4A); differently, after 48 weeks of therapy, an inverse correlation was found (Figure 4B). After adjustment for age (< or > = 40 years) and CD4 T cell count at baseline, PMN-MDSC frequency was still associated with CD4 T count >500 cells/mmc at T48 (Table 2). These data suggest that PMN-MDSC persistence may reflect a worse immune reconstitution.

**Table 2:** Multivariate logistic regression exploring factors associated with CD4 T cell >500 at 48 months.

	**OR**	**95% CI**		***p*-value**
CD4 (BL)	0.12	0.01	1.57	0.107
**Age, Years**				
<40	1.00			
≥40	0.85	0.73	0.99	0.0342
MDSC	0.78	0.62	0.99	0.038

We wondered whether PMN-MDSC may affect the commitment of CD34+ hematopoietic progenitor cells toward T cells differentiation. To this aim, we cultured purified CD34 stem cells from HD on OP9-DL1 stromal cells in the presence of IL-7, FLT3, and SCF to support the expansion of lymphoid progenitors. We found a reduction of early progenitor frequency when PMN-MDSC were added at a ratio of 1:3 (Figures 4C,D), suggesting that PMN-MDSC may interfere with T cell commitment of early hematopoietic progenitor cells. Moreover, to exclude that the decrease of CD34+Lin-CD38- cells was not due to PMN-MDSC-induced CD34 cell death, we evaluated the cell recovery after culture, and found no difference among CD34 alone and CD34 cultured with PMN-MDSC at 1:1 and 1:3 ratios (mean ± SD: 397500 ± 142916, 523750 ± 330842, 515000 ± 312250, respectively). This finding was confirmed by evaluating the induction of 7AAD on CD34+ cells when PMN-MDSC were added, corroborating that PMN-MDSC did not induce CD34+ cell death (Figure 4C).

### Plasmatic TRAIL and GM-CSF Level After ART

**Figure 5:**
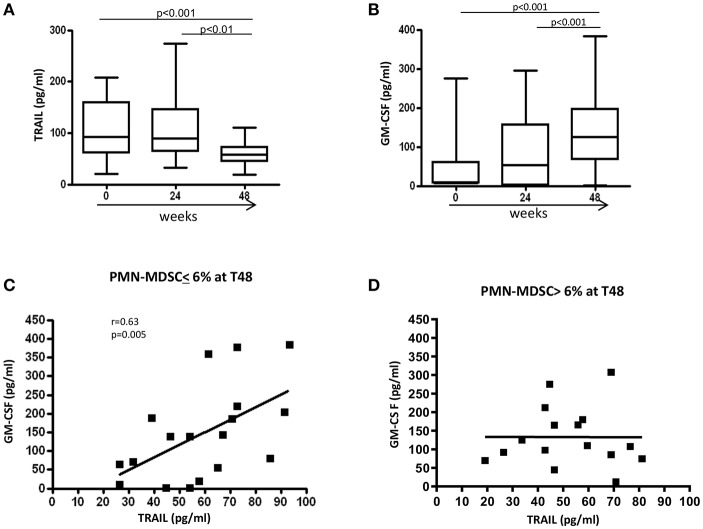
Modulation of factors involved in PMN-MDSC accumulation. Plasmatic level (pg/ml) of TRAIL **(A)** and GM-CSF **(B)** before and after 24 and 48 weeks of ART (*n* = 34). Results are shown as Box and Whiskers. The Friedman test with Dunn's multiple comparison post test was applied. Correlation between TRAIL and GM-CSF plasmatic levels after 48 weeks of therapy in patients with a PMN-MDSC frequency lower **(C)** (*n* = 18) and higher **(D)** (*n* = 16) than 6% at the same time point. The Spearman test was applied to evaluate correlations. *p* < 0.05 was considered statistically significant.

We recently reported that, in treatment-naïve PHI patients, PMN-MDSC frequency inversely correlated with plasmatic TRAIL level, while no correlation was found with the other inflammatory cytokines (17). We also showed that TRAIL was able to induce PMN-MDSC apoptosis, and GM-CSF inhibited the pro-apoptotic function of TRAIL. Herein, we evaluated the TRAIL plasmatic level during ART, and found that 48 weeks after ART initiation TRAIL significantly decreased compared to T0 (Figure 5A). Interestingly, an increase of GM-CSF was observed at T48 (Figure 5B). However, no correlation was found between PMN-MDSC frequency and TRAIL or GM-CSF levels (data not shown).

To further study the regulatory mechanisms that operate in patients with low (<6%) and high (>6%) PMN-MDSC frequency after 48 weeks of therapy, correlations were performed between TRAIL and GM-CSF at T48 in the two groups. We found a direct correlation between TRAIL and GM-CSF levels in patients with low PMN-MDSC frequency at T48 (Figure 5C). In contrast, such a correlation was not present in the group with high PMN-MDSC frequency (Figure 5D). These data suggest the loss of co-regulated expression of TRAIL and GM-CSF may induce uncontrolled PMN-MDSC expansion. We did not find any correlations between PMN-MDSC frequency and other pro-inflammatory cytokines (data not shown).

## Discussion

The expansion of different subsets of MDSC has been clearly demonstrated during HIV infection (14–16). However, contrasting data have been reported on the effect of ART on the modulation of MDSC frequency. We previously reported an increased frequency of PMN-MDSC in chronically HIV-infected patients regardless of ART (16). In line with these results, herein we demonstrated that PMN-MDSC frequency of PHI patients remained stably higher than HD during therapy. In contrast, Vollbrecht and colleagues showed a decrease of PMN-MDSC in 7 patients after 6 weeks of therapy (14). This paper is only partially in contrast with our data, in fact we also observed a transient decrease in PMN-MDSC frequency at 12 weeks of ART in patients with a high frequency of PMN-MDSC (>6%) before ART initiation. These data suggest that during the first weeks of treatment, PMN-MDSC may decrease, but afterwards they increased to a level comparable to T0. Accordingly, it has been recently reported that ART intensification in PHI and CHI has no effects on PMN-MDSC frequency, remaining higher than HD in both three- and five-drug ART regiments (23). Importantly, PMN-MDSC maintain their suppressive activity after 48 weeks of ART, thus contributing to the persistence of immune dysfunction. Altogether, the data demonstrate that the anti-HIV treatment, even if efficient in clearing the virus, is not able to induce a normalization of PMN-MDSC frequency after 48 weeks. We excluded that PMN-MDSC persistence could be associated with the residual viral load, confirming that after ART, HIV particles are not the main driver of PMN-MDSC maintenance.

Immune activation and inflammation are among the factors that may contribute to the HIV reservoir size (24) by favoring homeostatic or antigen-driven CD4 T_CM_ and T_TM_ cell proliferation latently infected with HIV (25). Given the high suppressive activity of MDSC on T cell proliferation and activation, our data suggest that PMN-MDSC might have an impact on viral reservoir size before starting therapy; however, after therapy a similar viral reservoir size was achieved, suggesting that the early ART initiation is able to reduce viral reservoir regardless of PMN-MDSC level.

MDSC frequency has been reported to be inversely correlated with CD4 T cell count in chronic HIV+ patients (14, 15). In contrast, we did not observe that correlation in treatment-naïve PHI patients (17), but after 48 weeks an inverse correlation was found, opening the question of whether and how PMN-MDSC may affect CD4 T cell recovery after ART. Our data show that *in vitro* PMN-MDSC may interfere with the expansion of the early hematopoietic progenitors, suggesting that PMN-MDSC have the capacity to modulate CD34+ hematopoietic stem cell commitment. The concept of MDSC functions other than direct immune suppression is not new, and include degradation of extracellular matrix, promotion of tumor cell invasion, angiogenesis, and formation of pre-metastatic niche (26, 27). Moreover, Chen and colleagues demonstrated that MDSC from myelodysplastic patients can inhibit CD34+ hematopoietic stem cell differentiation toward myeloid and erythroid lineages (28), confirming that MDSC may impact the stem cell compartment.

Long-term production and accumulation of inflammatory factors lead to local and systemic immunosuppression associated with MDSC accumulation [reviewed in (29)]. In particular, GM-CSF is an important growth factor that drives the accumulation and suppressive function of MDSC in both mice (30) and patients (31) with cancer. We recently reported that TRAIL is able to control PMN-MDSC frequency in PHI, while GM-CSF correlates with PMN-MDSC accumulation during chronic HIV infection (17). In the present report, we found that GM-CSF increased overtime in PHI under ART, and the balance between TRAIL and GM-CSF may regulate circulating PMN-MDSC frequency. TRAIL is a member of the TNF family able to induce apoptosis in target cells, including MDSC (32). We previously demonstrated that GM-CSF is able to abrogate the TRAIL-induced apoptosis of PMN-MDSC (17). Thus, we can speculate that, to better control PMN-MDSC accumulation, the increase of GM-CSF needs to be balanced by TRAIL. The reason why the GM-CSF level is raised during efficient therapy remains to be investigated.

In conclusion, we found that early ART initiation was not able to normalize PMN-MDSC frequency, and this persistence may have an impact on the HIV-specific T cell response, and on the hematopoietic stem cell compartment, too. Our data open new question on the effect of the long-term persistence of PMN-MDSC during HIV infection.

## Data Availability

All datasets generated for this study are included in the manuscript and/or the supplementary files.

## Ethics Statement

The study was approved by the Institutional Review Board of the INMI Lazzaro Spallanzani (ALPHA and SIREA studies) and signed written informed consent was obtained from all patients in accordance with the Declaration of Helsinki.

## Author Contributions

ASac and CA contributed to the conception and designed the study. CP and AM enrolled patients. CA, NT, AAm, IA, VB, and ASab performed the experiments. CA, NT, and ASac analyzed the cytofluorimetric data. EC, VB, RC, and GG contributed to analyzing data. ASac and PL performed the statistical analysis. ASac and CA wrote the draft of the manuscript. VB, RC, and AAn wrote sections of the manuscript. All authors contributed to manuscript revision, read and approved the submitted version.

### Conflict of Interest Statement

The authors declare that the research was conducted in the absence of any commercial or financial relationships that could be construed as a potential conflict of interest.
